# Placental Tissue Calcification and Its Molecular Pathways in Female Patients with Late-Onset Preeclampsia

**DOI:** 10.3390/biom14101237

**Published:** 2024-09-30

**Authors:** Miguel A. Ortega, Tatiana Pekarek, Diego De Leon-Oliva, Diego Liviu Boaru, Oscar Fraile-Martinez, Cielo García-Montero, Julia Bujan, Leonel Pekarek, Silvestra Barrena-Blázquez, Raquel Gragera, Patrocinio Rodríguez-Benitez, Mauricio Hernández-Fernández, Laura López-González, Raul Díaz-Pedrero, Ángel Asúnsolo, Melchor Álvarez-Mon, Natalio García-Honduvilla, Miguel A. Saez, Juan A. De León-Luis, Coral Bravo

**Affiliations:** 1 Department of Medicine and Medical Specialities, (CIBEREHD), Faculty of Medicine and Health Sciences, University of Alcalá, 28801 Alcala de Henares, Spain; tatianapekarek@gmail.com (T.P.); diegodleonoliva01@gmail.com (D.D.L.-O.); diego.boaru@edu.uah.es (D.L.B.); oscarfra.7@gmail.com (O.F.-M.); cielo.gmontero@gmail.com (C.G.-M.); mjulia.bujan@uah.es (J.B.); leonel.pekarek@gmail.com (L.P.); raquel.gragera@uah.es (R.G.); mademons@gmail.com (M.Á.-M.); natalio.garcia@uah.es (N.G.-H.); msaega1@oc.mde.es (M.A.S.); 2 Ramón y Cajal Institute of Sanitary Research (IRYCIS), 28034 Madrid, Spain; silvebarrena@gmail.com (S.B.-B.); laura.lgonzalez@uah.es (L.L.-G.); raul.diazp@uah.es (R.D.-P.); angel.asunsolo@uah.es (Á.A.); 3 Department of Nursing and Physiotherapy, Faculty of Medicine and Health Sciences, University of Alcalá, 28801 Alcala de Henares, Spain; 4 Department of Public and Maternal and Child Health, School of Medicine, Complutense University of Madrid, 28040 Madrid, Spain; prodriguezb@senefro.org (P.R.-B.); jaleon@ucm.es (J.A.D.L.-L.); cbravoarribas@gmail.com (C.B.); 5 Department of Obstetrics and Gynecology, University Hospital Gregorio Marañón, 28009 Madrid, Spain; 6 Health Research Institute Gregorio Marañón, 28009 Madrid, Spain; 7 Department of Nephrology, University Hospital Gregorio Marañón, 28009 Madrid, Spain; 8 Department of Surgery, Medical and Social Sciences, Faculty of Medicine and Health Sciences, University of Alcalá, 28801 Alcala de Henares, Spain; lmauricio.hernandez@uah.es; 9 Immune System Diseases-Rheumatology and Internal Medicine Service, University Hospital Prince of Asturias, Networking Research Center on for Liver and Digestive Diseases (CIBEREHD), 28806 Alcala de Henares, Spain; 10 Pathological Anatomy Service, University Hospital Gómez-Ulla, 28806 Alcala de Henares, Spain

**Keywords:** placenta, late-onset preeclampsia (LO-PE), calcification, placental villi, gene expression, tissue expression

## Abstract

Preeclampsia (PE) is a complex multisystem disease characterized by hypertension of sudden onset (>20 weeks’ gestation) coupled with the presence of at least one additional complication, such as proteinuria, maternal organ dysfunction, or uteroplacental dysfunction. Hypertensive states during pregnancy carry life-threatening risks for both mother and baby. The pathogenesis of PE develops due to a dysfunctional placenta with aberrant architecture that releases factors contributing to endothelial dysfunction, an antiangiogenic state, increased oxidative stress, and maternal inflammatory responses. Previous studies have shown a correlation between grade 3 placental calcifications and an elevated risk of developing PE at term. However, little is known about the molecular pathways leading to placental calcification. In this work, we studied the gene and protein expression of c-Jun N-terminal kinase (JNK), Runt-related transcription factor 2 (RUNX2), osteocalcin (OSC), osteopontin (OSP), pigment epithelium-derived factor (PEDF), MSX-2/HOX8, SOX-9, WNT-1, and β-catenin in placental tissue from women with late-onset PE (LO-PE). In addition, we employed von Kossa staining to detect mineral deposits in placental tissues. Our results show a significant increase of all these components in placentas from women with LO-PE. Therefore, our study suggests that LO-PE may be associated with the activation of molecular pathways of placental calcification. These results could be the starting point for future research to describe the molecular mechanisms that promote placental calcification in PE and the development of therapeutic strategies directed against it.

## 1. Introduction

Hypertensive disorders of pregnancy (HDPs) are a leading cause of maternal and perinatal morbidity and mortality worldwide, affecting up to 10% of pregnancies [1]. HDPs include preeclampsia (PE), chronic hypertension, pregnancy-induced hypertension, and superimposed preeclampsia [2,3]. PE is a life-threatening, progressive multisystem disease of pregnancy characterized by new-onset hypertension (systolic blood pressure ≥ 140 mmHg and diastolic blood pressure ≥ 90 mmHg) diagnosed after 20 weeks, often accompanied by proteinuria [4]. The global incidence of PE between 2002 and 2010 was estimated at 4.6% of all births [5]. Patients with PE are more susceptible to severe complications, such as eclampsia, hemorrhagic stroke, hemolysis, elevated liver enzymes and low platelet counts (HELLP syndrome), placental abruption, renal failure, and pulmonary edema [6,7]. After PE, women have an increased risk of mortality, particularly from stroke, Alzheimer’s disease, cardiovascular disease, and diabetes [8,9]. Newborns, on the other hand, are at increased risk of fetal distress, growth restriction, cardiovascular dysfunction, impaired cognitive ability, and neurodevelopmental outcomes [8,9]. Therefore, it is necessary to understand the pathophysiology of PE to treat it and reduce its impact on maternal and neonatal health.

The placenta is a central structure in pregnancy, supporting fetal growth for nine months. It performs pleiotropic functions, such as maternal–fetal exchange; endocrine activity; mechanical, chemical, and immunological barrier formation; and determination of maternal–fetal programming [10]. This organ is formed by the invasion of the trophoblast into the endometrium, which remodels the uterine spiral arteries to form the trophoblast lacunae with maternal blood, which will become the intervillous spaces [11]. Placental villi are formed from the chorionic plate. They have an outer layer of syncytiotrophoblast (STB) on the outside and cytotrophoblast (CTB) on the inside, and inside are the fetal vessels that join to form the umbilical vessels surrounded by placental connective and fibrinoid tissue [12]. Through the STB and CTB bathed in maternal blood, the exchange of substances between the embryo and the mother takes place.

The placenta plays a key role in the onset and progression of PE, as the disease only manifests when there is or has recently been a placenta, and delivery represents the only “cure”. There are different criteria for the classification of PE. The International Society for the Study of Hypertension in Pregnancy (ISSHP) distinguishes three types of PE according to gestational age at the time of clinical presentation: preterm (<37 weeks’ gestation), term (≥37 weeks’ gestation), and postpartum (diagnosed after delivery). However, its classification into early- and late-onset preeclampsia (EO-PE and LO-PE, respectively) according to whether clinical symptoms appear before or after 34 weeks of pregnancy, respectively, is also useful for research, as it appears to reflect substantial differences in etiology [13]. EO-PE is related to alterations in placentation, resulting in abnormal placental development and function, especially in spiral artery remodeling and trophoblast invasion [14]. In contrast, LO-PE is more frequent and is mainly associated with compression of placental villi when there is insufficient space for the larger placenta at the end of pregnancy and syncytiotrophoblastic senescence associated with premature placental aging [15].

The general model of PE development establishes two stages. The first pathophysiological events detected in PE are abnormal placental implantation, characterized by defective remodeling of the uterine spiral artery and superficial invasion of the extravillous trophoblast, resulting in a 50–70% decrease in uteroplacental perfusion [16,17]. Hypoperfusion leads to placental hypoxia, ischemia/reperfusion injury, and oxidative stress [18]. These changes are responsible for the onset of a maternal inflammatory response, apoptosis, and an alteration in the factors released by the placenta during pregnancy with systemic implications [18,19,20]. Stressed STB releases anti-angiogenic factors, such as the soluble form of tyrosine kinase-1 (sFLT1) or soluble endoglin (sEng), and decreases the production of nitric oxide and vascular endothelial growth factor (VEGF), leading to endothelial dysfunction [18,21]. It also produces proinflammatory cytokines, reactive oxygen species (ROS), and extracellular vesicles (EVs) [22,23,24]. The main histopathological findings come from the entry of turbulent flows into the intervillous space that ruptures the anchoring villi [25]. These include decidual arteriopathy, fibrinoid necrosis, accelerated villous maturation, or villous infarcts [19,26].

Calcification plays a key role in tissue stiffness, especially in the human placenta. Pathological deposition of calcium salts can occur in any soft tissue in the body, although the best studied occur in blood vessels, kidneys, brain, lungs, tendons, and breast cancer [27]. Three main types of calcifications can be distinguished: physiological, dystrophic, and metastatic. Physiological calcification is responsible for the formation of bones and teeth. Dystrophic calcification occurs in damaged tissues, related to ischemia and necrosis, appearing as amorphous deposits [28]. On the other hand, metastatic calcification occurs in healthy tissues when high serum calcium concentrations are reached [29].

Placental calcification is the deposition of calcium phosphate minerals in the placental tissue itself [30]. It occurs in the placenta in both healthy and pathological states and is related to gestational age, although the degree is quite variable [31]. The etiology is unknown, and it is found both in the maternal decidua and in the placental villous tree. A few studies have described both dystrophic and metastatic calcifications [32,33]. A systematic review by Schiffer et al. described increased placental thickness and increased presence of placental lacunae and placental calcifications in pregnancies affected by the placental syndrome, as observed in antepartum placentas by ultrasound [34]. Despite the clinical importance of PE, the molecular mechanisms that cause placental tissue calcification in the context of PE are largely unknown, and the associations between them are poorly defined.

Our previous studies on the placentas of LO-PE patients have confirmed that there are signs of oxidative stress, lipid peroxidation, ferroptosis, and inflammasome activation [35,36]. Different signaling pathways have been described that influence and regulate the calcification process, including calcium signaling, Wnt/β-catenin, BMP/Smad, and Notch [37,38,39]. The c-Jun N-terminal kinase (JNK) is a multifunctional kinase that regulates various physiological and pathological processes, including the regulation of the response to events that alter cellular homeostasis [40]. Runt-related transcription factor 2 (RUNX2) regulates several osteoblast genes by binding to the promoter, including type I collagen, osteopontin (OSP), osteocalcin (OSC), and bone sialoprotein [41]. RUNX2, OSC, and OSP are osteogenic proteins that have also been described in vascular calcification [42,43]. In addition, previous studies have found an increase in pigment epithelium-derived factor (PEDF) in placentas from women with venous insufficiency related to placental calcification [44]. The transcription factors MSX2/HOX8 and SOX9 are involved in osteogenesis and chondrogenesis processes [45,46]. Finally, the Wnt/β-catenin signaling pathway is of great importance both in embryonic development and in adult tissue homeostasis, and its activation has been demonstrated in pathological calcification processes [47,48]. However, the involvement of these signaling pathways and their connection in placental calcification is unknown, both in healthy conditions and in different pathological contexts of pregnancy, including PE. We hypothesized that LO-PE increases the number of calcium salt deposits in placental villi and alters gene and tissue expression of biomarkers of calcification-related molecular pathways. Therefore, this study aimed to investigate gene and tissue level expression of the biomarkers of tissue calcification, inflammation, and angiogenesis; JNK, RUNX2, OSC, OSP, PEDF, MSX-2/HOX8, SOX-9, WNT-1, and β-catenin; as well as calcium deposits, identifying the types, in placental villi of LO-PE patients by histopathological study, using immunohistochemistry (IHC), real-time quantitative PCR (RT-qPCR), and von Kossa staining.

## 2. Patients and Methods

### 2.1. Study Design and Participants

An observational, analytical, prospective study nested in a cohort was designed. A total of 111 women in their third trimester of pregnancy (32 weeks) whose pregnancy was followed at the Hospital Central de la Defensa de Madrid were recruited (Table 1). Sixty-eight patients were clinically diagnosed with LO-PE. Likewise, 43 healthy patients with no history of PE were studied as a control group (HC). LO-PE was diagnosed according to the American College of Obstetricians and Gynecologists (ACOG) Clinical Practice Guidelines criteria for “Gestational Hypertension and Preeclampsia” in patients with PE who had some of the following severity criteria: systolic blood pressure ≥ 160 mmHg and/or diastolic blood pressure ≥ 110 mmHg confirmed at 15 min; proteinuria ≥ 2 g measured in 24 h urine or estimated by urine protein/creatinine ratio; oliguria ≤ 500 mL/24 h or diuresis rate < 0.5 mL/kg/h for 2 h; renal insufficiency with serum creatinine > 1.1 mg/dL or twice the serum creatinine value in the absence of other renal disease; neurologic or visual disturbances, such as severe headache that does not subside with analgesics, blurred vision, diplopia, or amaurosis; acute pulmonary edema or cyanosis; pain in the epigastrium or right hypochondrium; liver dysfunction with transaminase levels elevated to twice the normal value; hematologic disorders such as thrombocytopenia (<100,000/mm^3^), disseminated intravascular coagulation, or hemolysis; and placental involvement with fetal manifestations, such as intrauterine growth restriction, abnormal umbilical artery Doppler results, and fetal death. In this study, we considered the presence of a serum creatinine level higher than 1.1 mg/dL as a criterion for the severity of preeclampsia. A total of 43 pregnant women without identified diseases were also included as healthy controls (HC). This study was conducted following good clinical practice guidelines; the postulates of the Declaration of Helsinki (2013), the Oviedo Convention (1997), and the ethical principles of autonomy, beneficence, and non-maleficence; and was approved by the Clinical Research Ethics Committee of the Hospital Universitario Central de la Defensa-UAH (LIB 12/2022 of 30 September 2022).

### 2.2. Sample Collection and Processing

After delivery, placental biopsies were taken from patients with LO-PE and HC. Five placental fragments per sample, including mixed cotyledons, were collected with a scalpel. These fragments were then divided into two separate sterile tubes: one with RNAlater^®^ solution (Ambion; Thermo Fisher Scientific, Inc., Waltham, MA, USA) and one with minimal essential medium (MEM; Thermo Fisher Scientific, Inc., Waltham, MA, USA) supplemented with 1% antibiotic/antimycotic (streptomycin, amphotericin B and penicillin; Thermo Fisher Scientific, Inc.). Placenta samples were then processed in a class II laminar flow hood (Telstar AV 30/70 Müller 220 V 50 MHz; Telstar; Azbil Corporation, Chiyoda-ku, Tokyo, Japan) under sterile conditions. To preserve the samples for further processing and investigation of gene expression, they were immersed in 1 mL of RNAlater^®^ and stored at −80 °C.

To remove erythrocytes, samples preserved in MEM were washed and rehydrated five times in MEM without antibiotics. Each sample was then cut into 2 cm pieces using a second scalpel and fixed in F13 solution (60% ethanol, 20% methanol, 13% distilled water, and 7% polyethylene glycol) following established protocols [49]. After embedding the samples in paraffin using molds, 5 µm thick sections were obtained using an HM 350 S rotary microtome (Thermo Fisher Scientific, Inc., Waltham, MA, USA) and transferred to 10% poly-L-lysine treated glass slides after placing them in a warm water bath. These samples were then used for tissue and gene expression analysis.

### 2.3. Immunohistochemistry and Histological Visualization

Samples were initially deparaffinized with xylol, followed by ethanol at decreasing concentrations (100%, 96%, and 70%), and then hydrated with distilled water. Subsequently, IHC was performed using an avidin–biotin complex and avidin peroxidase for the identification of antigen/antibody reactions using established techniques [50] (Detailed immunohistochemistry (IHC) procedure can be found at Supplementary Materials). Placental tissues were washed three times with 1x PBS at an interval of five minutes. Non-specific binding sites were then blocked using 3% bovine serum albumin (BSA) diluted in PBS at room temperature (RT) for 30 min. In all sections, endogenous peroxidase was blocked by incubating the tissue in 3% hydrogen peroxide (H_2_O_2_, PanReac AppliChem, 121076.111, Castellar del Vallès (Barcelona), Spain) for 10 min [20,35]. Samples were then incubated with the primary antibody for 90 min, followed by overnight incubation at 4 °C with 3% BSA blocker (cat. no. 37525; Thermo Fisher Scientific, Inc., Waltham, MA, USA). The next day, placenta samples were incubated for 90 min at RT with a biotin-conjugated secondary antibody previously diluted in PBS (Table 2). Subsequently, the avidin–peroxidase conjugate ExtrAvidin^®^–Peroxidase (Sigma-Aldrich; Merck KGaA, St. Louis, MO, USA) was added for one hour at RT (1:200 dilution in PBS). Finally, a chromogenic kit with diaminobenzidine (DAB) substrate (cat. no. SK-4100; Maravai LifeSciences, San Diego, CA, USA) was prepared immediately before use (5 mL distilled water; four drops of DAB; two drops of hydrogen peroxide and two drops of buffer) to determine the level of protein expression.

Peroxidase chromogenic substrate was used for 15 min at room temperature to visualize the positive signal as brown staining. For each immunohistochemical experiment, sections of the same tissue were used as negative controls, where the primary antibody incubation was replaced by incubation in PBS and blocking solution. For contrast, nuclei were stained with Carazzi’s hematoxylin for 5 to 15 min. Samples were rinsed in tap water for 10 min and then mounted in Plasdone aqueous polymer. Table 2 provides detailed information on the antibodies used in our study as well as protocol guidelines. Histological analysis was performed with a Zeiss Axiophot light microscope (Carl Zeiss, Oberkochen, Germany). Each placental specimen was subjected to investigation of five different sections and ten random fields by two independent histologists. IHC expression was assessed according to the immunoreactivity score (IRS), with positivity defined as a mean stained area equal to or greater than 5% in the analyzed sample, as established in previous studies [51]. The percentage of positive cells was calculated using the following formula: % Positive cells = No. Cells^+^/(No. Cells^−^ + No. Cells^+^) × 100. The intensity of immunostaining was graded on a scale of 0 to 3, corresponding to minimal (≤25%), moderate (25–65%), and intense (≥65–100%) staining levels.

### 2.4. Study of Gene Expression

Using real-time quantitative polymerase chain reaction (RT-qPCR), the amount of cDNA present in each sample for the gene of interest was quantified. First, RNA extraction was performed using the guanidinium thiocyanate–phenol–chloroform technique [52]. This method facilitates the analysis of mRNA expression levels of specific genes.

Reverse transcription was performed to generate complementary DNA (cDNA) from RNA samples at a 50 ng/µL concentration. Four µL of each sample was mixed with 4 µL of oligo-dT solution at 0.25 µg/µL (Thermo Fisher Scientific, Inc., Waltham, MA, USA) and the RNA was denatured at 65 °C for 10 min in a dry bath (AccuBlock, Labnet International Inc., Cary, NJ, USA). Samples were then cooled on ice and treated with a reverse transcription mixture containing 2.8 µL of 5× first strand buffer (250 mM Tris-HCl at pH 8.3; 375 mM KCl: 15 mM MgCl_2_), 1 µL of retrotranscriptase enzyme, 2 µL of 10 mM deoxyribonucleotide triphosphate, 2 µL of 0 1 M dithiothreitol, 1.7 µL of DNase- and RNase-free water, and 0.5 µL of RNase inhibitor (RNase Out), all from Thermo Fisher Scientific, Inc., Waltham, MA, USA.

Reverse transcription was performed using the G-Storm GS1 thermal cycler (G-Storm Ltd., Middlesbrough, UK). Samples were incubated at 37 °C for 1 h and 15 min for cDNA synthesis. The temperature was then raised to 70 °C and held for 15 min to denature the retrotranscriptase, which was gradually reduced to 4 °C. Negative reverse transcription was performed by replacing M-MLV retrotranscriptase with DNase- and RNase-free water to ensure the absence of genomic DNA contamination in the RNA samples. The cDNA synthesized at room temperature was diluted 1:20 in DNase- and RNase-free water and stored at −20 °C until further use.

To design specific primers for the selected genes, the online tools Primer-BLAST and AutoDimer were used (Table 3) [53,54]. The constitutively expressed gene *TATA-box binding protein* (TBP) served as a control to normalize the results. Gene expression units were quantified as relative mRNA concentrations. Using the relative standard curve method, RT-qPCR was performed on a StepOnePlus™ system (Applied Biosystems, Foster City, CA, USA; Thermo Fisher Scientific, Inc., Waltham, MA, USA). The reaction mixture was prepared with 5 µL of sample; diluted 1:20; and mixed with 10 µL of iQ™ SYBR^®^ Green Supermix (Bio-Rad Laboratories, Inc., Hercules, CA, USA), 1 µL each of the direct and reverse primers, and 3 µL of DNase- and RNase-free water, which were added to a 96-well MicroAmp^®^ plate (Applied Biosystems; Thermo Fisher Scientific, Inc.). Thermocycling conditions consisted of an initial denaturation at 95 °C for 10 min, followed by denaturation at 95 °C for 15 s, annealing at variable temperatures depending on the melting temperature of the primer pairs for 30 s and elongation at 72 °C for 1 min, in 40–45 cycles. Subsequently, a dissociation curve was developed at 95 °C for 15 s, 60 °C for 1 min, 95 °C for 15 s and 60 °C for 15 s. Fluorescence detection was performed at the end of each cycle and at various points along the dissociation curve. Data were incorporated into a standard curve generated by serial dilutions of various samples, including constitutive TBP expression, following the manufacturer’s guidelines. This RT-qPCR procedure was repeated twice for all placental tissue samples to ensure consistency of results.

### 2.5. Von Kossa Stain for Calcium Deposits

Von Kossa staining was applied to placenta samples to recognize calcium deposits (brown-black), differentiating them from the rest of the tissue (red). The following steps were followed: the samples were stained with silver nitrate for 20 min, rinsed in 5% sodium hyposulfite for 15 min, and then rinsed in tap water. The samples were then stained with safranin for 1 min, dehydrated in 96% alcohol for 3 min, dehydrated again in 100% alcohol for 5 min, and rinsed with xylol for 10 min. Finally, the samples were mounted with Cytoseal^™^. Under the light microscope, the calcified areas appear black because of silver reduction.

### 2.6. Statistical Analysis

Data were analyzed with GraphPad Prism^®^ v6.0 (GraphPad, Inc., San Diego, CA, USA). The Mann–Whitney U-test was applied, and the Pearson χ^2^ test was used. Results were expressed using median, maximum, and minimum values. Significance thresholds were set at *p* < 0.05 (*), *p* < 0.01 (**), and *p* < 0.001 (***).

## 3. Results

### 3.1. Placentas from Women with LO-PE Show JNK Activation

**Figure 1:**
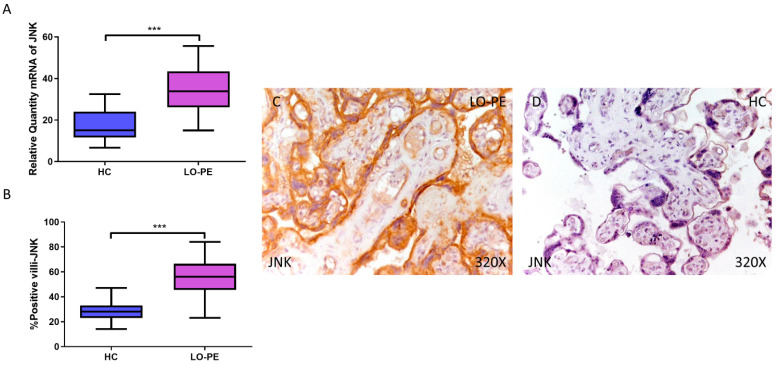
Increased gene and protein expression of JNK in placental villi of patients with late-onset preeclampsia (LO-PE). (**A**) Relative amount of mRNA coding for JNK in LO-PE patients and healthy controls (HC). (**B**) Percentage of immunoreactivity for JNK expression in placental villi of LO-PE and HC group. (**C**,**D**) Photomicrographs showing JNK immunostaining in LO-PE and HC placental villi. *n* (HC) = 43; *n* (LO-PE) = 68. *p* < 0.001 (***).

First, our RT-qPCR findings demonstrate a significant increase in JNK gene expression in the placental tissue of pregnant women with LO-PE (LO-PE = 33.834 [15.016–56.616]; HC = 15.087 [6.652–32.459], *** *p* < 0.0001, Figure 1A). Immunohistochemical analysis of tissue expression of JNK in placental villi revealed significantly increased JNK expression in LO-PE patients compared to HC (LO-PE = 56,000 [23,000–84,000]; HC = 28,000 [14,000–47,000], *** *p* < 0.0001, Figure 1B). JNK protein expression was predominantly localized in the STB and CTB of the villi, and in the fetal vessel walls (Figure 1C,D).

### 3.2. Placentas from Women with LO-PE Show Elevated Osteogenesis Markers RUNX2, OSC, and OSP

**Figure 2:**
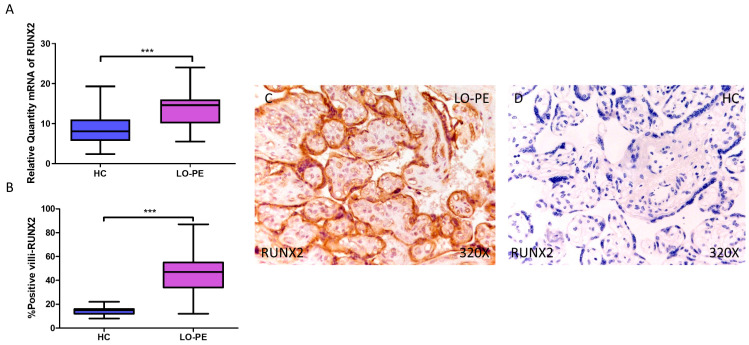
Increased gene and protein expression of RUNX2 in placental villi of patients with late-onset preeclampsia (LO-PE). (**A**) Relative amount of mRNA coding for RUNX2 in LO-PE patients and healthy controls (HC). (**B**) Percentage of immunoreactivity for RUNX2 expression in placental villi of LO-PE and HC group. (**C**,**D**) Photomicrographs showing RUNX2 immunostaining in the placental villi of LO-PE and HC. *n* (HC) = 43; *n* (LO-PE) = 68. *p* < 0.001 (***).

Next, we determined the gene expression of the osteogenesis markers RUNX2, OSC, and OSP. Our RT-qPCR results show a statistically significant increase in RUNX2 gene expression in placental tissue from pregnant women with LO-PE (LO-PE = 14.616 [5.497–24.032]; HC = 8.062 [2.361–19.300], *** *p* < 0.0001, Figure 2A). Immunohistochemical analysis of RUNX2 expression in placental villi revealed increased RUNX2 expression in LO-PE patients compared to healthy controls (LO-PE = 47,000 [12,000–87,000]; HC = 15,000 [8000–22,000], *** *p* < 0.0001, Figure 2B). RUNX2 protein expression was observed in both STB and CTB of villi, as well as in other connective tissue cells (Figure 2C,D).

**Figure 3:**
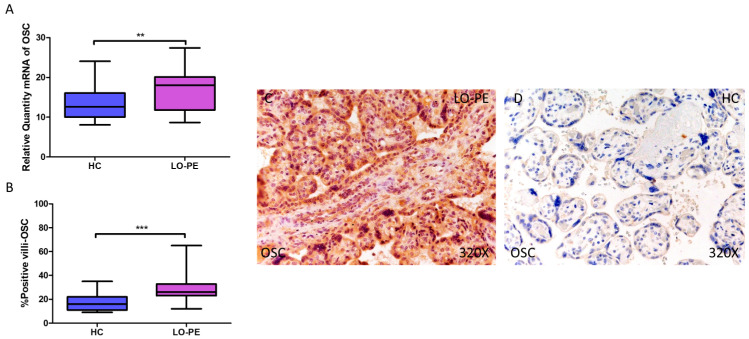
Increased gene and protein expression of OSC in placental villi of patients with late-onset preeclampsia (LO-PE). (**A**) Relative amount of mRNA coding for OSC in patients with LO-PE and healthy controls (HC). (**B**) Percentage of immunoreactivity for OSC expression in placental villi of LO-PE and HC group. (**C**,**D**) Photomicrographs showing OSC immunostaining in LO-PE and HC placental villi. *n* (HC) = 43; *n* (LO-PE) = 68. *p* < 0.01 (**); *p* < 0.001 (***).

Simultaneously, a significant increase in OSC expression was determined, both at the mRNA level (LO-PE = 18,065 [8652–27,415]; HC = 12,606 [8065–24,065], ** *p* = 0.0015, Figure 3A) and protein level (LO-PE = 26,000 [12,000–65,000]; HC = 16,000 [9000–35,000], *** *p* < 0.0001, Figure 3B). Tissue expression of OSC was strongly manifested along the placental villi of LO-PE-affected women compared to HC (Figure 3C,D).

**Figure 4:**
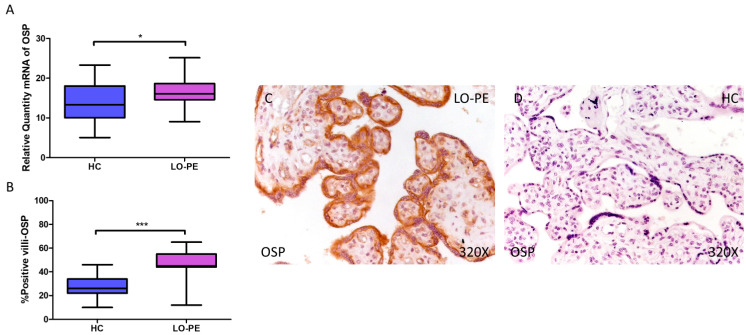
Increased gene and protein expression of OSP in placental villi of patients with late-onset preeclampsia (LO-PE). (**A**) Relative amount of mRNA coding for OSP in patients with LO-PE and healthy controls (HC). (**B**) Percentage of immunoreactivity for OSP expression in placental villi of LO-PE and HC group. (**C**,**D**) Photomicrographs showing OSP immunostaining in LO-PE and HC placental villi. *n* (HC) = 43; *n* (LO-PE) = 68. *p* < 0.05 (*); *p* < 0.001 (***).

Finally, regarding OSP expression, a higher relative amount of OSP mRNA was quantified by RT-qPCR in LO-PE women compared to HC women (LO-PE = 16.065 [9.065–25.156]; HC = 13.303 [5.062–23.312], * *p* = 0.0119, Figure 4A). IHC analysis of placental villi showed increased OSC expression in LO-PE patients (LO-PE = 45,000 [12,000–65,000]; HC = 26,000 [10,000–46,000], *** *p* < 0.0001, Figure 4B). Visualization of the samples by light microscopy showed increased OSP expression along the STB and CTB of the chorionic villi (Figure 4C,D).

### 3.3. Placentas from Women with LO-PE Show Increased Expression of the Angiogenesis Inhibitor PEDF

**Figure 5:**
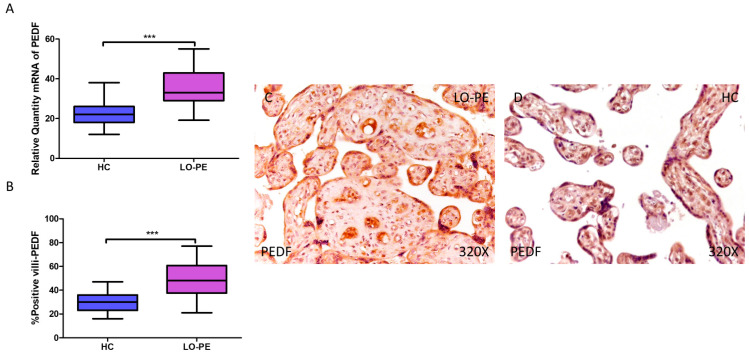
Increased gene and protein expression of PEDF in placental villi of patients with late-onset preeclampsia (LO-PE). (**A**) Relative amount of mRNA coding for PEDF in patients with LO-PE and healthy controls (HC). (**B**) Percentage of immunoreactivity for PEDF expression in placental villi of LO-PE and HC group. (**C**,**D**) Photomicrographs showing PEDF immunostaining in LO-PE and HC placental villi. *n* (HC) = 43; *n* (LO-PE) = 68. *p* < 0.001 (***).

PEDF gene expression was significantly higher in women with LO-PE compared to healthy patients (LO-PE = 33.016 [19.156–55.065]; HC = 22.065 [12.016–38.065], *** *p* < 0.0001, Figure 5A). IHC revealed significantly higher expression of PEDF in placentas from LO-PE patients (LO-PE = 48,000 [21,000–77,000]; HC = 30,000 [16,000–47,000], *** *p* < 0.0001, Figure 5B). PEDF was highly expressed throughout the chorionic villi of placentas from LO-PE women, predominantly in the STB, CTB, and fetal vessels, whereas in the HC group, it was less strongly distributed within the villi (Figure 5C,D).

### 3.4. Placentas from Women with LO-PE Show Activation of the Transcription Factors MSX2/HOX8 and SOX9

**Figure 6:**
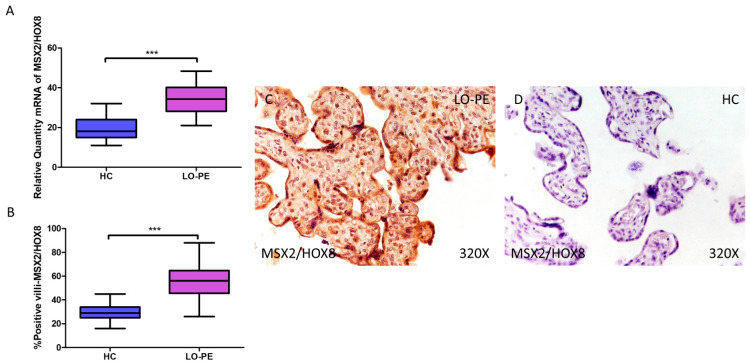
Increased gene and protein expression of MSX2/HOX8 in placental villi of patients with late-onset preeclampsia (LO-PE). (**A**) Relative amount of mRNA coding for MSX2/HOX8 in patients with LO-PE and healthy controls (HC). (**B**) Percentage of immunoreactivity for MSX2/HOX8 expression in placental villi of LO-PE and HC group. (**C**,**D**) Photomicrographs showing MSX2/HOX8 immunostaining in LO-PE and HC placental villi. *n* (HC) = 43; *n* (LO-PE) = 68. *p* < 0.001 (***).

**Figure 7:**
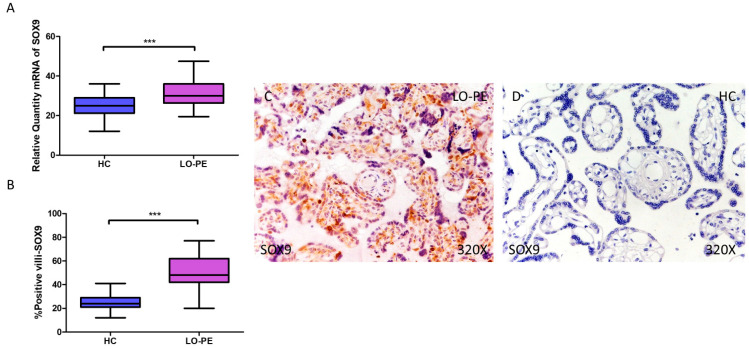
Increased gene and protein expression of SOX9 in placental villi of patients with late-onset preeclampsia (LO-PE). (**A**) Relative amount of mRNA coding for SOX9 in LO-PE patients and healthy controls (HC). (**B**) Percentage of immunoreactivity for SOX) expression in placental villi of LO-PE and HC group. (**C**,**D**) Photomicrographs showing SOX9 immunostaining in LO-PE and HC placental villi. *n* (HC) = 43; *n* (LO-PE) = 68. *p* < 0.001 (***).

RT-qPCR analysis of mRNA levels revealed a significant increase in MSX2/HOX8 gene expression in the placentas of women with LO-PE compared to those of the HC group (LO-PE = 34.238 [21.012–48.354]; HC = 18.066 [11.017–32.062], *** *p* < 0.0001, Figure 6A). By IHC, a significant increase in IRS score for MSX2/HOX8 was established in placental villi of women with LO-PE (LO-PE = 56,000 [26,000–88,000]; HC = 29,000 [16,000–45,000], *** *p* < 0.0001, Figure 6B). High levels of expression were detected in the STB and CTB, as well as in interior cells (Figure 6C,D). SOX9 gene expression was found to be significantly higher in the LO-PE group of patients (LO-PE = 30.093 [19.455–47.456]; HC = 25.012 [12.065–36.065], *** *p* < 0.0001, Figure 7A). Likewise, IHC revealed significantly higher expression in the extracellular matrix of placental villi in women with LO-PE compared to women in the HC group (LO-PE = 48,000 [20,000–77,000]; HC = 24,000 [12,000–41,000], *** *p* < 0.0001, Figure 7B–D).

### 3.5. Placentas from Women with LO-PE Show an Increase in the Canonical Wnt Pathway

**Figure 8:**
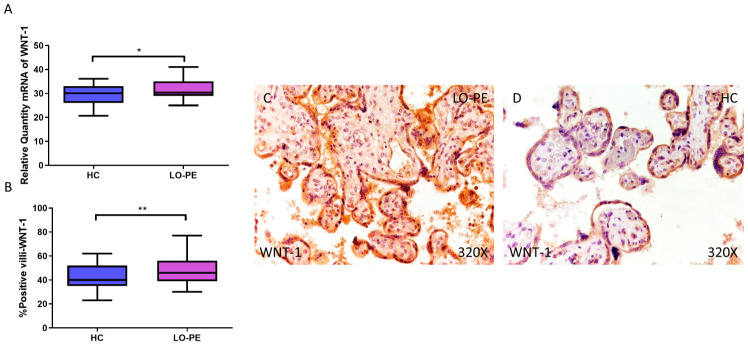
Increased gene and protein expression of WNT-1 in placental villi of patients with late-onset preeclampsia (LO-PE). (**A**) Relative amount of mRNA coding for WNT-1 in LO-PE patients and healthy controls (HC). (**B**) Percentage of immunoreactivity for WNT-1 expression in placental villi of LO-PE and HC group. (**C**,**D**) Photomicrographs showing WNT-1 immunostaining in LO-PE and HC placental villi. *n* (HC) = 43; *n* (LO-PE) = 68. *p* < 0.05 (*); *p* < 0.01 (**).

Finally, the expression of WNT-1 and β-catenin signaling was analyzed. Relative quantification of WNT-1 mRNA by RT-qPCR was found to be significantly higher in the LO-PE group compared to the HC group (LO-PE = 30.359 [25.015–41.012]; HC = 30.120 [20.655–36.103], * *p* = 0.0265, Figure 8A). The HI established by IHC of placentas from women with LO-PE was significantly higher (LO-PE = 46,000 [30,000–77,000]; HC = 40,000 [23,000–62,000], ** *p* = 0.0047, Figure 8B). The protein was expressed in the STB, CTB, and endothelium of fetal capillaries (Figure 8C,D).

**Figure 9:**
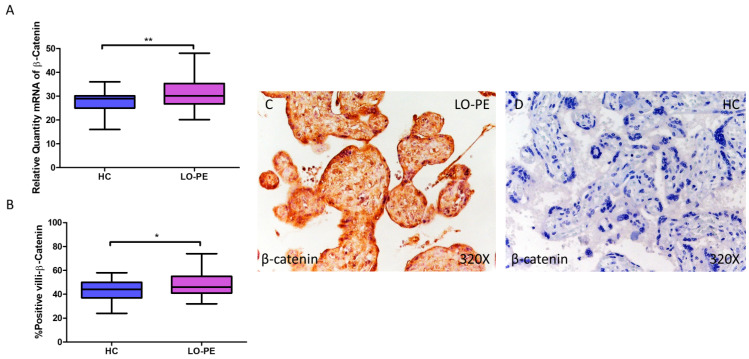
Increased gene and protein expression of β-catenin in placental villi of patients with late-onset preeclampsia (LO-PE). (**A**) Relative amount of mRNA coding for β-catenin in patients with LO-PE and healthy controls (HC). (**B**) Percentage of immunoreactivity for β-catenin expression in placental villi of LO-PE and HC group. (**C**,**D**) Photomicrographs showing β-catenin immunostaining in LO-PE and HC placental villi. *n* (HC) = 43; *n* (LO-PE) = 68. *p* < 0.05 (*); *p* < 0.01 (**).

β-catenin gene expression quantified by RT-qPCR was found to be significantly higher in placentas from women with LO-PE compared to the HC group (LO-PE = 30.126 [20.145–48.056]; HC = 29.032 [16.032–36.016], ** *p* = 0.0023, Figure 9A). IHC revealed significantly higher tissue expression of β-catenin in placental villi of LO-PE patients (LO-PE = 46,000 [32,000–74,000]; HC = 44,000 [24,000–58,000], ** *p* = 0.0226, Figure 9B). Tissue expression of β-catenin was found throughout the chorionic villi, predominantly in the STB and CTB.

### 3.6. Increased Presence of Calcifications in the Placental Tissue of Patients with LO-PE

**Figure 10:**
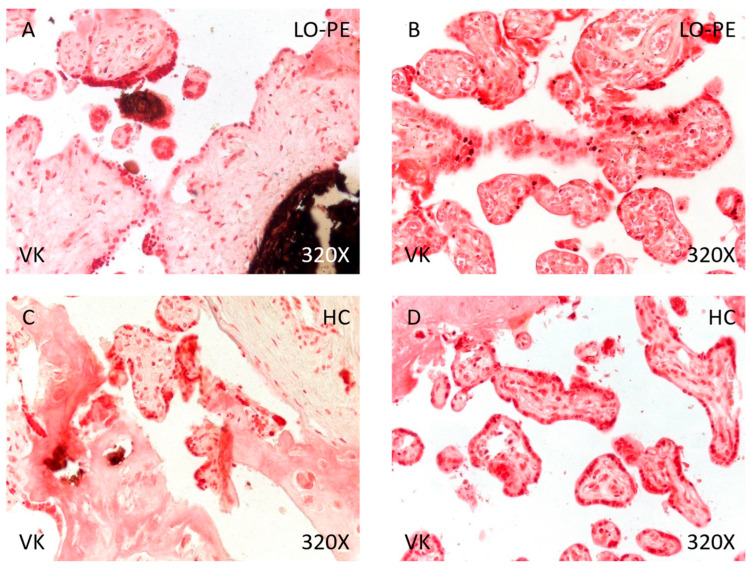
Von Kossa staining of placental villi from patients with late-onset preeclampsia (LO-PE) and healthy controls (HC). (**A**,**C**). Photomicrographs showing dystrophic calcifications in patients with LO-PE and HC. (**B**,**D**). Photomicrographs showing metastatic calcifications in patients with LO-PE and HC.

Finally, analysis of placental tissue calcifications showed that the percentage of calcium deposits was significantly higher in women with LO-PE than in the HC group (89.71% vs. 48.84%, χ^2^ (1, *n* = 111) = 22.80, *** *p* < 0.0001, Table 4). Regarding the type of calcifications, women with LO-PE had a higher percentage of metastatic than dystrophic calcifications (62.30% vs. 37.70%, Figure 10A,B). Conversely, the percentage of dystrophic calcifications was higher in the HC group (52.38% vs. 47.62%, Figure 10C,D).

## 4. Discussion

PE is a life-threatening disease of pregnancy characterized by new-onset hypertension after 20 weeks of pregnancy (systolic pressure ≥140 mm Hg and/or diastolic pressure ≥90 mm Hg). PE is responsible for more than 500,000 fetal and neonatal deaths and more than 70,000 maternal deaths [55]. Although calcifications are frequently observed in both healthy and diseased placentas, both their mechanisms and their impact on clinical outcomes are unknown, especially in the context of PE [56,57,58]. In this work, we demonstrated abnormal expression of JNK, RUNX2, OSC, OSP, PEDF, MSX-2/HOX8, SOX-9, WNT-1, and β-catenin and increased deposition of calcium salts in placental villi of women with LO-PE. These findings confirm our hypothesis, indicating that LO-PE is associated with specific changes in placental calcification and the regulation of key molecular pathways in this process.

Our results are in agreement with previous studies that have also identified a relationship between PE and the increased presence of placental calcifications, as well as in other pathologies of pregnancy affecting the placenta, including pregnancy-induced hypertension, intrauterine growth restriction, and chorangiosis [34,59,60,61]. In addition, deficiency of the phosphate transporters Slc20a1 and Slc20a2 in the human placenta results in both placental calcifications and PE [62,63]. Furthermore, ultrasound identification of grade III placental calcifications by Grannum classification could help to identify pregnancies at risk of pregnancy-induced proteinuric hypertension and intrauterine growth restriction [64]. On the other hand, our findings demonstrate that LO-PE induces specific changes in the expression of important mediators in calcification signaling pathways, suggesting possible molecular mechanisms responsible for placental calcification.

First, placental villi from women with LO-PE show gene and protein overexpression of JNK compared to the HC group. This increase in JNK can be explained as the initiation of a cellular response to syncytiotrophoblastic stress caused by hypoxia and ischemia/reperfusion damage [65,66,67]. Both ischemia and hypoxia in the trophoblast line BeWo increase JNK via LEP and FLT1, respectively [68]. On the other hand, metformin has shown beneficial effects in the prevention of PE [69]. This drug activates AMP-activated protein kinase (AMPK), which inhibits the inflammatory response during hypoxia and reoxygenation through modulation of the JNK-mediated NF-κB pathway [65,70]. Therefore, the activation of JNK would be an adaptive response to the stress caused by hypoxia and the possible decrease in the protective signaling of AMPK.

Likewise, we demonstrated the increase in the osteogenesis markers RUNX2, OSC, and OSP in the placental villi of women with LO-PE. RUNX2 is a transcription factor that regulates the expression of several osteoblastic genes, including OSP and OSC, type I collagen, and bone sialoprotein, and for which a role in vascular calcification has been proposed in recent years [42,71]. RUNX2 also promotes the invasion of extravillous trophoblasts through the expression of matrix metalloproteinases 2 and 9 [72]. In addition, *miR-628-3p* is an inhibitor of RUNX2 that is found to be elevated in the blood of women who subsequently develop PE [73]. In mesenchymal stem cells (MSCs), the long non-coding RNA (lncRNA) HOTAIRM1 has been shown to promote osteogenesis in the same cells by controlling RUNX2 expression mediated by JNK/AP-1 signaling [74]. Their proposed model is based on the phosphorylation of c-Jun by JNK and targets the RUNX2 promoter, where it recruits the p300 acetyltransferase to activate the expression of RUNX2, which will initiate osteogenesis [75]. In addition, hypoxia-inducible factor 1 (HIF1), elevated in PE, is a positive modulator of RUNX2 through physical and functional interactions that promote angiogenesis and metabolic reprogramming to promote osteoblastogenesis and osteogenesis [76,77,78]. These effects could also take place in placental tissue under conditions of hypoxia that promote PE, initiating calcification pathways. Interestingly, recent studies have shown that HIF2 is a negative regulator of osteoblastogenesis, counteracting the effects of BMP2 and RUNX2 [79]. This effect is due, at least in part, to the transcription factor SOX9, which is also elevated in our samples of placentas with calcifications in cases of LO-PE.

OSC is a bone protein containing γ-carboxyglutamic acid present in the bone matrix, the most abundant non-collagenous organic component, secreted by osteoblasts. OSC expression in the placenta has also been described to play an important role in gestational diabetes mellitus [80]. It is involved in vascular calcification, through the regulation of vascular smooth muscle cell mitochondrial activity via WNT/β-catenin, which corresponds with our results in placental tissue [43,81]. Bone morphogenic protein 2 (BMP2) has been shown to induce osteoblast differentiation through the expression of OSC regulated by RUNX2 and ATF6 [41]. On the other hand, OSP is a multifunctional glycoprotein involved in both physiological and pathological calcification and immuno-inflammation in various pathological processes [82,83]. OSP is involved in implantation and placentation, promoting trophoblast invasion, which are key early steps in the establishment of a healthy pregnancy [84,85]. Plasma OSP concentrations are increased in PE with extensive endothelial injury [86]. Research by Xia et al. also identifies changes in the expression of OSP and its receptor, integrin ανβ3, in the PE placenta but downregulated [87]. Therefore, although the involvement of OSP in PE is demonstrated, the underlying mechanisms need to be investigated to fully explain these results. Regarding calcification, it has classically been attributed to a role in inhibiting ectopic calcification [88,89]. In response to vascular calcification damage, OSP promotes cell adhesion, proliferation, migration, and survival [90]. Recently, OSP has been shown to play a role as an anti-inflammatory mediator in patients with vascular calcification by promoting this phenotype in macrophages and inhibiting their differentiation into osteoclasts [91]. These results could be transferable to our own if this function could be demonstrated in Hofbauer cells, the placental resident macrophage population. Also, SPARC (secreted protein acidic and rich in cysteine), called osteonectin or BM-40, which serves as an adhesive between collagen and hydroxyapatite crystals, has been described as altered in placentas of women with PE and intrauterine growth restriction in both cytotrophoblastic cells and syncytiotrophoblasts [92].

Overall, our results suggest that the activation of RUNX2 and its downstream effectors in the placental tissue of LO-PE patients constitutes a bivalent response. On the one hand, the RUNX2-OSC axis would initiate placental calcification, while on the other hand, the RUNX2-OSP axis would initiate a protective counter-response to the former. However, this hypothesis should be studied in depth in future research to confirm the activation and functioning of these proposed pathways.

The increase in PEDF in calcified placental villi of women with LO-PE may be explained by several mechanisms related to the regulation of angiogenesis and the response to syncytiotrophoblastic stress. Our previous studies have demonstrated the upregulation of VEGF and PEDF in the placenta of women with lower limb venous insufficiency during pregnancy and their involvement in villous calcification [44]. In late pregnancy, trophoblast-produced PEDF levels increase, in combination with VEGF, to achieve precise control of fetoplacental angiogenesis [93]. In the hypoxic context of LO-PE, increased PEDF is an adaptive response to restrict vascular overgrowth and maintain trophoblast cell biological functions, and thus placental homeostasis [94]. On the other hand, it has been shown that PEDF promotes osteoblastic differentiation and mineral deposition via ERK/AKT in MSCs [95]. Thus, the observed correlation between elevated PEDF expression and the presence of calcium deposits in the placentas of women with LO-PE also suggests that PEDF may be facilitating calcification, in response to cell damage and tissue remodeling.

MSX2/HOX8 is a crucial transcription factor for cranial bone development and SOX9 is a master regulator of cartilage differentiation. Both are co-expressed in calcified vessels together with their target genes alkaline phosphatase, bone sialoprotein, osteocalcin, and collagen II [45,96,97]. MSX2/HOX8 plays important roles in early placental development such as promoting trophoblast invasion and maintaining the undifferentiated phenotype of trophoblast stem cells [98,99]. Defects in MSX2 activity may correspond to placental pathologies, such as PE. Two pathways have been described by which MSX2/HOX8 promotes vascular calcification: the E3 ligase MDM2 [100] and Wnt signaling [101,102]. On the other hand, SOX9 is a known transcription factor involved in chondrogenic differentiation, inhibiting the osteogenic function of RUNX2 [46]. In the context of LO-PE, where the placenta is under hypoxic conditions, the co-expression of MSX2/HOX8 and SOX9 reflects an adaptive response that could enhance the differentiation of trophoblast cells towards osteochondrogenic phenotypes, facilitating the formation of mineral deposits as a compensatory mechanism that attempts to maintain tissue integrity under stressful conditions, but which will ultimately impact on both maternal and fetal health.

**Figure 11:**
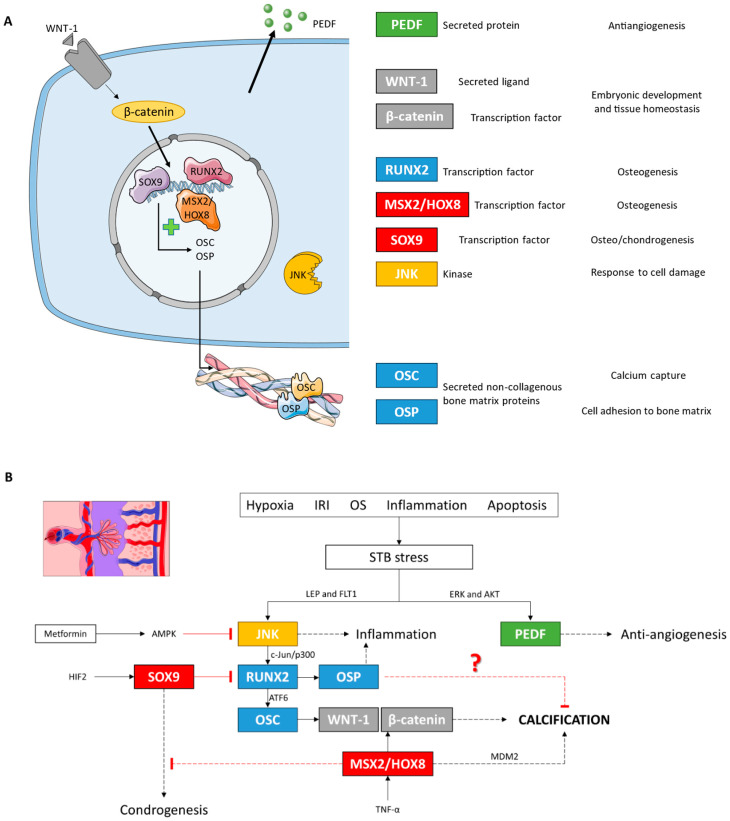
(**A**) Descriptive figure of the localization and function of the different molecules analyzed in this study. (**B**) Molecular signaling model of placental calcification under LO-PE conditions based on our results and studies in other cell lines. IRI: ischemia/reperfusion injury; OS: oxidative stress.

Finally, the expression of WNT-1 and β-catenin signaling proteins, the canonical Wnt pathway, was also increased in the placental villi of women with LO-PE. This pathway is necessary for proper embryonic development and maintaining adult tissue homeostasis [47]. Therefore, the Wnt pathway plays a crucial role in different processes of pregnancy such as implantation, decidualization, and placental differentiation [103,104,105]. Abnormal activation of Wnt signaling has been demonstrated in PE, probably due to alterations in trophoblast cell differentiation and function under stress conditions [106,107,108]. The involvement of Wnt signaling in vascular calcification has also been extensively demonstrated [109,110]. In addition, the canonical Wnt pathway can also activate the transcription factor RUNX2 in promoting osteogenesis and in osteosarcoma [111,112], while the non-canonical route activates JNK [113,114,115]. This body of evidence suggests that WNT-1 and β-catenin activation, in agreement with the rest of the markers, is an adaptive response to stress conditions, facilitating placental tissue calcification and stabilization (Figure 11).

Although calcifications were found in both the control and LO-PE groups, activation of all these pathways could be implicated in the significant increase in calcifications in the placental villi of women with LO-PE. This corresponds with previous studies that have suggested that hypoxia is responsible for placental calcifications [116]. Others also justify the use of ultrasound and three-dimensional power Doppler technology and computational analysis of placental calcifications to assess placental, and therefore fetal, status in pregnancies with complications such as PE or intrauterine growth restriction [34,60]. Regarding the type of calcifications, a higher proportion of metastatic calcifications was observed in women with LO-PE compared to dystrophic calcifications, which could reflect systemic alterations in calcium metabolism in LO-PE patients. However, this is difficult to justify as patients with PE show lower serum calcium levels than healthy patients, and the World Health Organization recommends dietary calcium supplementation during pregnancy as a preventive strategy for PE, being of special interest in low-resource countries [117,118]. In addition, perhaps the altered blood flow in the intervillous spaces of the placenta would manifest this type of calcifications, as we have already demonstrated in patients with venous insufficiency of the lower extremities [44]. The dystrophic calcifications correspond with various histopathological studies that identify necrosis in EP placentas and that hypoxia favors STB necrosis [19,26,119]. However, we cannot explain why there is a higher percentage of such calcifications in healthy patients than in LO-PE. Further studies with a larger number of samples are needed to corroborate our results and to investigate the possible underlying mechanisms.

We propose the following steps to be taken to advance this line of research: Firstly, it would be useful to carry out functional studies to investigate how the alteration in JNK, RUNX2, OSC, OSP, PEDF, MSX-2/HOX8, SOX-9, WNT-1, and β-catenin contributes to placental calcification. To this end, cell cultures of different trophoblast lines could be established and subjected to hypoxia to replicate the syncytiotrophoblastic stress present in PE. In this regard, we consider it equally important to study the role of EVs, both of maternal and placental origin, their content, and their impact on histopathological changes in placental tissue. In addition, possible therapeutic interventions that could normalize the expression of these factors and reduce calcification should be investigated to achieve a significant clinical impact. Also, it would be interesting to extend the study to different cohorts of patients, e.g., comparing EO-PE vs. LO-PE, to validate our findings and explore inter-individual variations and possible modulating factors. Finally, we propose the approach of longitudinal studies to follow the progression of placental calcification and its impact on maternal and fetal wellbeing.

Finally, our study has several important limitations that should be considered and addressed in future research. Among them, we highlight the limited sample size of calcifications, due to the difficulty in finding calcifications and the variable prevalence, ranging from 3.8% to 23.7% [118]. In addition, plasma levels of different biomarkers of PE, especially the placental released angiogenic factors sFLT1 and sEng [119], and serum levels of calcium salts that may contribute to metastatic calcifications could not be measured. Thus, further work should be carried out to overcome these limitations.

## 5. Conclusions

LO-PE is associated with abnormal expression of proteins involved in pathological calcification, inflammation, and angiogenesis. By RT-qPCR and IHC, we showed that LO-PE is associated with altered expression of JNK, RUNX2, OSC, OSP, PEDF, MSX2/HOX8, SOX9, WNT-1, and β-catenin proteins. The significant increase in calcium salt deposition in placental villi could be linked to specific changes in placental calcification associated with LO-PE. We described an increase in calcium deposition, both dystrophic and metastatic, in placentas affected by LO-PE. The activation of different molecular pathways and calcification could be a cellular response to hypoxia, inflammation, and oxidative stress characteristic of PE. These processes could be cellular adaptation mechanisms to maintain the anatomofunctional integrity of the placenta under adverse conditions. Lastly, there is a need for further research focused on exploring in depth the specific mechanisms of placental calcification in LO-PE and developing effective therapeutic interventions.

## Figures and Tables

**Table 1:** Clinical and demographic characteristics of patients included in this study and healthy controls. HC = healthy controls; LO-PE = late-onset preeclampsia; *n* = number of patients evaluated; SD = standard deviation; NS = not significant. *p* < 0.05 (*); *p* < 0.001 (***).

	HC (*n* = 43)	LO-PE (*n* = 68)	*p*-Value
Average age (years) ± SD	31,348 ± 5117	29,015 ± 4816	* *p* = 0.0154
Nulliparous (total number) *n* (%)	14 (32.56)	53 (77.94)	*** *p* < 0.0001
Average gestational age (weeks) ± SD	39,069 ± 1486	38,627 ± 1434	NS
Cesarean sections (total number) *n* (%)	8 (18.60)	15 (22.06)	NS
Placenta weight (g) mean ± SD	500,977 ± 65,331	370,254 ± 61,647	*** *p* < 0.0001

**Table 2:** Information on primary and secondary antibodies used in immunohistochemistry.

Antigen	Species	Dilution	Provider	Protocol Specifications
**JNK**	Rabbit	1:250	Abcam (ab124956) Cambridge, UK	0.1% Triton in PBS, 10 min before incubation with blocking solution.
**RUNX2**	Goat	1:100	Santa Cruz (sc-8566) Dallas, TX, USA	0.1% Triton in PBS, 10 min before incubation with blocking solution.
**OSC**	Goat	1:50	Santa Cruz (sc-18319) Dallas, TX, USA	0.1% Triton in PBS, 10 min before incubation with blocking solution.
**OSP**	Mouse	1:50	Santa Cruz (sc-73631) Dallas, TX, USA	0.1% Triton in PBS, 10 min before incubation with blocking solution.
**PEDF**	Mouse	1:500	Abcam (ab115489) Cambridge, UK	Citrate buffer in heat (pH = 6).
**MSX2/HOX8**	Rabbit	1:100	Abcam (ab190070) Cambridge, UK	Sodium citrate 10 mM pH = 6 before incubation with blocking solution.
**SOX9**	Mouse	1:250	Abcam (ab76997) Cambridge, UK	Sodium citrate 10 mM pH = 6 before incubation with blocking solution.
**WNT-1**	Rabbit	1:50	Abcam (ab63934) Cambridge, UK	0.1% Triton in PBS, 10 min before incubation with blocking solution.
**β-catenin**	Mouse	1:100	Abcam (ab231305) Cambridge, UK	No protocol specifications
**IgG (Rabbit)**	Mouse	1:1000	Sigma-Aldrich (RG96/B5283) St. Louis, MO, USA	No protocol specifications
**IgG (Cabra)**	Mouse	1:100	Sigma-Aldrich (GT34/B3148) St. Louis, MO, USA	No protocol specifications
**IgG (Ratón)**	Goat	1:300	Sigma-Aldrich (F2012/045K6072) St. Louis, MO, USA	No protocol specifications

**Table 3:** Primers used for RT-qPCR: sequences, forward (Fwd) and reverse (Rev), and binding temperatures (T).

Gene	Sequence Fwd (5′ → 3′)	Sequence Rev (5′ → 3′)	T (°C)
**TBP**	TGCACAGGAGCCAAGAGTGAA	CACATCACAGCTCCCCACCA	60
**JNK**	TGCCTGACAAAAGCAAGCAATCA	TCTGAGTCAGCGTTTTCCTTGT	61
**RUNX2**	CGCCTCACAAACAACCACAG	TCACTGTGCTGAAGAGGCTG	59
**OSC**	ATGAGAGCCCTCACACTCCT	CTTGGACACAAAGGCTGCAC	61
**OSP**	AGCAGAATCTCCTAGCCCCA	ACGGCTGTCCCAATCAGAAG	57
**PEDF**	GTGTGCAGGCTTAGAGGGACTA	ATGCAGAGGAGTAGCACCAG	50
**MSX2/HOX8**	ATTCAGAAGATGGAGCGGCG	ATATGTCCTCCTACTCCTGCCC	59
**SOX9**	GGAAGTCGGTGAAGAACGGG	CAAGGTCGAGTGAGCTGTGT	57
**WNT-1**	TGCGCTTCCTCATGAACCTT	TGCTAGCGAGTCTGTTTGGG	59
**β-catenin 1**	GGAGGAAGGTCTGAGGAGCA	AGGCTCCAGAAGCAGTCATC	60

**Table 4:** Percentages of patients with calcium deposits and types (metastatic and dystrophic) and *p*-value results of Pearson χ^2^ test. HC = healthy controls; LO-PE = late-onset preeclampsia; *n* = number of cases. *p* < 0.001 (***).

	HC (*n* = 43)	LO-PE (*n* = 68)	*p*-Value
**No calcification % (*n*)**	51.16% (22)	10.29% (7)	*** *p* < 0.0001
**Calcification % (*n*)**	48.84% (21)	89.71% (61)	
**Metastatic % (*n*)**	47.62% (10)	62.30% (38)	
**Dystrophic % (*n*)**	52.38% (11)	37.70% (23)	

## Data Availability

The data used to support the findings of the present study are available from the corresponding author upon request.

## Supplementary Materials

The following supporting information can be downloaded at: https://www.mdpi.com/article/10.3390/biom14101237/s1, File S1: Detailed immunohistochemistry (IHC) procedure.
