# Supplementary material for: Placental Tissue Calcification and Its Molecular Pathways in Female Patients with Late-Onset Preeclampsia
Source: Biomolecules. 2024 Sep 30;14(10):1237. doi: 10.3390/biom14101237 (PMC11506500; doi:10.3390/biom14101237)
Supplement: Supplementary file 1 [file biomolecules-14-01237-s001.zip › biomolecules-3119194-supplementary.pdf]

**Supplementary material. Detailed immunohistochemistry (IHC) procedure**

The samples used for immunohistochemical studies are processed similarly to those used for optical microscopy analysis. Once embedded in paraffin and sectioned into 5 µm-thick slices via microtomy, they are deparaffinized and hydrated. For all immunohistochemical studies, sections from the same tissue are used as negative controls, where incubation with the primary antibody is replaced by incubation in a blocking solution.

The detection of the antigen-antibody reaction is performed using the ABC method (avidin-biotin complex), employing peroxidase as the chromogen, following this protocol:

1. Wash the samples with 1x PBS, for three cycles of 5 minutes each.
2. Block non-specific binding sites using 3% BSA (bovine serum albumin) in PBS, for 30 minutes at room temperature.
3. Incubate with the primary antibody (according to the manufacturer's specifications) diluted in 3% BSA and PBS, overnight at 4°C.
4. Wash with PBS, for three cycles of 5 minutes each.
5. Incubate with the biotin-conjugated secondary antibody (according to the manufacturer's specifications) diluted in PBS, for 1 hour and 30 minutes at room temperature.
6. Wash with PBS, for three cycles of 5 minutes.
7. Incubate with the avidin-peroxidase conjugate (ExtrAvidin®-Peroxidase, Sigma-Aldrich, St. Louis, MO, USA) for 1 hour at room temperature. Dilution: 1/200 in PBS.
8. Wash with PBS, for three cycles of 5 minutes each.
9. A. Develop by incubating with the chromogenic substrate diaminobenzidine (DAB Kit, SK-4100) (Vector, Burlingame, CA, USA). The chromogenic substrate is prepared immediately before development.

To prepare the substrate:

- 5 mL of distilled water
- 2 drops of buffer
- 4 drops of DAB.
- 2 drops of hydrogen peroxide.

This technique results in a brown-colored stain.

10. Counterstain the nuclei using Carazzi's hematoxylin for 5-15 minutes.
11. Wash with running water for 10 minutes.
12. Mount using an aqueous medium with Plasdone.
